# Genomic Data Reveal Conserved Female Heterogamety in Giant Salamanders with Gigantic Nuclear Genomes

**DOI:** 10.1534/g3.119.400556

**Published:** 2019-08-22

**Authors:** Paul M. Hime, Jeffrey T. Briggler, Joshua S. Reece, David W. Weisrock

**Affiliations:** * Department of Biology, University of Kentucky, Lexington, KY 40506,; † Missouri Department of Conservation, Columbia, MO 65201, and; ‡ Department of Biology, California State University, Fresno, CA 93740

**Keywords:** Amphibian, W chromosome, ddRADseq, *Cryptobranchus*, *Andrias*, Genetics of Sex

## Abstract

Systems of genetic sex determination and the homology of sex chromosomes in different taxa vary greatly across vertebrates. Much progress remains to be made in understanding systems of genetic sex determination in non-model organisms, especially those with homomorphic sex chromosomes and/or large genomes. We used reduced representation genome sequencing to investigate genetic sex determination systems in the salamander family Cryptobranchidae (genera *Cryptobranchus* and *Andrias*), which typifies both of these inherent difficulties. We tested hypotheses of male- or female-heterogamety by sequencing hundreds of thousands of anonymous genomic regions in a panel of known-sex cryptobranchids and characterized patterns of presence/absence, inferred zygosity, and depth of coverage to identify sex-linked regions of these 56 gigabase genomes. Our results strongly support the hypothesis that all cryptobranchid species possess homologous systems of female heterogamety, despite maintenance of homomorphic sex chromosomes over nearly 60 million years. Additionally, we report a robust, non-invasive genetic assay for sex diagnosis in *Cryptobranchus* and *Andrias* which may have great utility for conservation efforts with these endangered salamanders. Co-amplification of these W-linked markers in both cryptobranchid genera provides evidence for long-term sex chromosome stasis in one of the most divergent salamander lineages. These findings inform hypotheses about the ancestral mode of sex determination in salamanders, but suggest that comparative data from other salamander families are needed. Our results further demonstrate that massive genomes are not necessarily a barrier to effective genome-wide sequencing and that the resulting data can be highly informative about sex determination systems in taxa with homomorphic sex chromosomes.

How are organisms able to produce distinct sexes from a single genome? In many cases, an individual’s sex is determined genetically by one or more sex-determining loci which can result in an evolutionary cascade of profound genomic consequences, including degradation of the chromosome bearing the sex-determining locus (Steinemann and Steinemann 1998; Charlesworth *et al.* 2005; Graves 2006; Bachtrog 2013), accumulation of sexually antagonistic loci on sex chromosomes (Rice 1984; van Doorn and Kirkpatrick 2007; Innocenti and Morrow 2010; Charlesworth *et al.* 2014), genome-wide changes in dosage compensation (Mank 2009; Gu and Walters 2017; Marin *et al.* 2017; Rupp *et al.* 2017), and the buildup of reproductive isolation during population divergence and speciation (Sæther *et al.* 2007; Kitano *et al.* 2009; Lima 2014; Bracewell *et al.* 2017; O’Neill and O’Neill 2018). In mammals and birds, one of the two sex chromosomes is typically heavily degenerated and evident cytogenetically via classical karyotypic analyses (*i.e.*, heteromorphic sex chromosomes) (Graves 2006; Bachtrog 2013). However, in other lineages, sex chromosomes are much harder to diagnose and appear to be much more labile over evolutionary timescales. In contrast to well-studied mammalian and avian systems, many organisms exhibit homomorphic sex chromosomes that cannot be diagnosed cytogenetically, making it difficult to determine sex chromosome homology among species.

Homomorphic sex chromosomes predominate in amphibians, with multiple transitions among XY and ZW systems of sex determination (Bull 1983; Hillis and Green 1990; Schmid *et al.* 1991; Hayes 1998; Wallace *et al.* 1999; Schmid and Steinlein 2001; Ogata *et al.* 2003; Eggert 2004; Nakamura 2009; Furman and Evans 2016; Miura 2017; Pennell *et al.* 2018; Jeffries *et al.* 2018). Variation in sex chromosomes has been documented between species (Stöck *et al.* 2011; Brelsford *et al.* 2013; Roco *et al.* 2015; Sessions *et al.* 2016), and even within populations of the same species (Rodrigues *et al.* 2014), suggesting that in some amphibians, sex chromosomes may be especially labile. Furthermore, there appears to be little bias in the direction of transitions between XY and ZW systems (Evans *et al.* 2012; Pennell *et al.* 2018), with transition rates between homomorphic and heteromorphic sex chromosomes in amphibians appearing roughly equal [but see Hillis and Green (1990)].

Sex chromosome systems appear to be labile across salamanders, with multiple hypothesized transitions among XY and ZW systems (Sessions 2008), although data are only available from seven of the ten recognized families (Figure 1). The superfamily Cryptobranchoidea comprises the Asiatic salamanders (family Hynobiidae) and the giant salamanders (family Cryptobranchidae). Hynobiids are thought to possess a ZW system of female-heterogametic sex determination (Kuro-o *et al.* 1998, 2002; Ikebe *et al.* 2005). The family Cryptobranchidae is also thought to possess a ZW system (Sessions *et al.* 1982). The family Sirenidae is suspected to have a ZW system of female heterogamety (León and Kezer 1974). The remaining seven salamander families comprise the superfamily Salamandroidea, of which at least three families have species with XY systems. If the putative ZW systems of Cryptobranchidae, Hynobiidae, and Sirenidae were shown to be homologous, that would imply that the ancestral mode of sex determination in salamanders is ZW and that ZW to XY transitions occurred along branches leading to XY species in Salamandroidea. The family Ambystomatidae has a ZW system sex determination (Smith and Voss 2009; Keinath *et al*. 2017, 2018) and relatively homomorphic sex chromosomes. The family Proteidae is comprised of two deeply divergent genera, *Proteus* and *Necturus*, both of which possess an XY system of male heterogamety (Sessions 2008; Sessions *et al*. 2016). While *Proteus* has homomorphic sex chromosomes, possibly resulting from an X-Y translocation, *Necturus* has heteromorphic XY sex chromosomes (Sessions *et al*. 2016). The two most speciose families of salamanders, Salamandridae and Plethodontidae, each contain genera with XY and ZW sex determination systems and genera with homomorphic and heteromorphic sex chromosomes (Sessions 2008), implying that there have been additional transitions between male- and female-heterogamety within these lineages.

**Figure 1:**
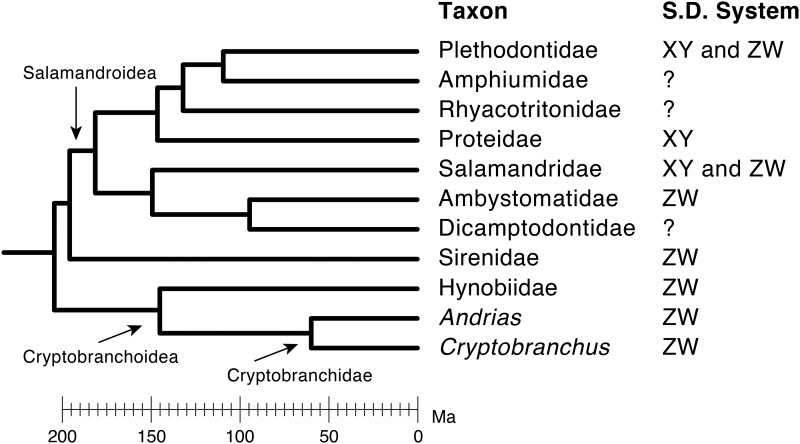
Family-level relationships among extant salamanders are depicted, along with the distribution of XY (male heterogametic) and ZW (female heterogametic) sex determination (S.D.) systems. Families for which S.D. mode is not known are indicated with a question mark. Divergence times are based on Kumar *et al.* (2017) and units are in millions of years before present (Ma). Information about sex determination systems is from León and Kezer (1974), Ikebe *et al.* (2005), Sessions (2008), Smith and Voss (2009), and this study.

As is typical of most salamanders (Keinath *et al.* 2015; Elewa *et al.* 2017; Evans *et al.* 2018; Nowoshilow *et al.* 2018; Smith *et al.* 2019), cryptobranchids have gigantic nuclear genomes over 56 Gb (Gregory 2019), nearly 18 times larger than the human genome. These huge genomes are putatively not due to polyploidization, but instead may be the result of ancient expansions of retrotransposons (Sun and Mueller 2014) and increases in intergenic content in the genome (Smith *et al.* 2009), potentially related to low metabolic rates among salamanders. Cryptobranchids are long-lived salamanders exhibiting delayed sexual maturity (4–7 years), and males and females are only easily distinguishable during a narrow annual time window during the breeding season when males have a swollen cloaca (Nickerson and Mays 1973). *Cryptobranchus* and *Andrias* are both thought to possess a ZW system of female heterogamety (Sessions 2008), though there are conflicting karyotypic reports in the literature. Sessions *et al.* (1982) used cytogenetic techniques to identify a ZW system in Cryptobranchidae, but karyotypic analyses in *Andrias* by Zhu *et al.* (2002), appear to have found a heteromorphic XY system. Furthermore, it is not known whether *Cryptobranchus* and *Andrias* share a conserved system of sex determination or homologous sex chromosomes, and genomic data are likely required to resolve these uncertainties (Hu *et al.* 2019).

Whole genome sequencing (WGS) methods are now widely used to identify sex-linked loci in non-model organisms (Bachtrog 2013; Keinath *et al.* 2018). However, the expense and other complications associated with applying WGS to organisms with extremely large genomes has prevented widespread use of these methods in salamanders. Reduced representation genomic approaches, such as restriction site-associated DNA sequencing (RADseq) (Baird *et al.* 2008; Baxter *et al.* 2011; Peterson *et al.* 2012), have become a powerful alternative to WGS to identify sex chromosome-associated loci and to distinguish male- from female-heterogametic systems (Palaiokostas *et al.* 2013; Brown *et al.* 2016; Fowler and Buonaccorsi 2016; Gamble 2016; Lambert *et al.* 2016; Brelsford *et al.* 2017; Nielsen *et al.* 2018; Stovall *et al.* 2018; Jeffries *et al.* 2018; Hu *et al.* 2019). RADseq-based approaches involve inherent tradeoffs among the numbers of loci sequenced, their depths of sequencing coverage, and the numbers of individuals that can be effectively multiplexed on current high-throughput sequencing platforms (Weisrock *et al.* 2018). Double digest RADseq (ddRADseq) protocols (Peterson *et al.* 2012) are particularly well-suited to identify sex chromosome-associated regions in massive genomes because they can be readily tailored to target different numbers of loci in a given species by varying either of the restriction enzymes (REs) used and/or the particular fragment size-selection window (Figure S1). By comparing patterns of presence and absence of loci between known males and known females, researchers can bioinformatically identify candidate sex-linked markers which can be further validated by the polymerase chain reaction (PCR). Explicit statistical models are also now available to identify sets of candidate sex-linked RAD loci sequenced from known-sex individuals (Gamble *et al.* 2015; Stovall *et al.* 2018).

Sex-linked markers also provide important conservation genetic resources for endangered species. *Cryptobranchus* was historically widespread in streams and rivers across eastern and central North America (Nickerson and Mays 1973), but wild populations are now in sharp decline (Wheeler *et al.* 2003; Pitt *et al.* 2017). Both species of *Andrias* are also in peril (Ota 2000; Wang *et al.* 2004; Chen *et al.* 2018; Turvey *et al.* 2018), and all three currently recognized cryptobranchid species are threatened or endangered throughout their ranges (Hammerson and Phillips 2004; Kaneko and Matsui 2004; Liang *et al.* 2004). *In situ* and *ex situ* conservation efforts are underway to stabilize wild populations and establish captive breeding populations for eventual re-release (Ettling *et al.* 2017; Murphy and Gratwicke 2017; Kenison and Williams 2018). But, these conservation programs are hindered by the difficulty of accurately determining sex for cryptobranchids. Ultrasound and laparoscopy have been employed to diagnose sex in *Cryptobranchus* and *Andrias* (Kramer *et al.* 1983; Roth and Obringer 2003; Li *et al.* 2010; Kraus *et al.* 2017), but these techniques are not universally effective, require expert interpretation, and, in the case of laparoscopy, are invasive. Serum calcium level differences may also distinguish females from males (Nickerson and Mays 1973), but there are numerous advantages to a genetic sex assay including effectiveness across all age classes and seasons and the ability to analyze banked tissue samples. Recently, Hu *et al.* (2019) used ddRADseq to identify W-linked loci in *Andrias davidianus*, indicating an ability for these methods to identify sex-linked markers, but further questions remain, including the conservation of ZW sex determination systems in this salamander family, and the robustness of the application of ddRADseq in large genomes.

We expand on previous investigations of the evolution of sex determination systems in cryptobranchid salamanders by first performing ddRADseq in 20 known-sex *Cryptobranchus* individuals. We used bioinformatic analyses of these data to compare regions of the genome assembled from males and females and to test expectations of the mutually exclusive hypotheses of male or female heterogamety. Our *in silico* analyses produced sets of candidate male- and female-specific loci which we then validated by PCR in known-sex individuals from several divergent populations of *Cryptobranchus* and in both species of *Andrias* (Figure 2). Our results are consistent with sex chromosome stasis in the salamander family Cryptobranchidae and suggest that female heterogamety has been conserved in this lineage for over 60 million years, in stark contrast to the rapid turnovers observed in many other amphibian lineages.

**Figure 2:**
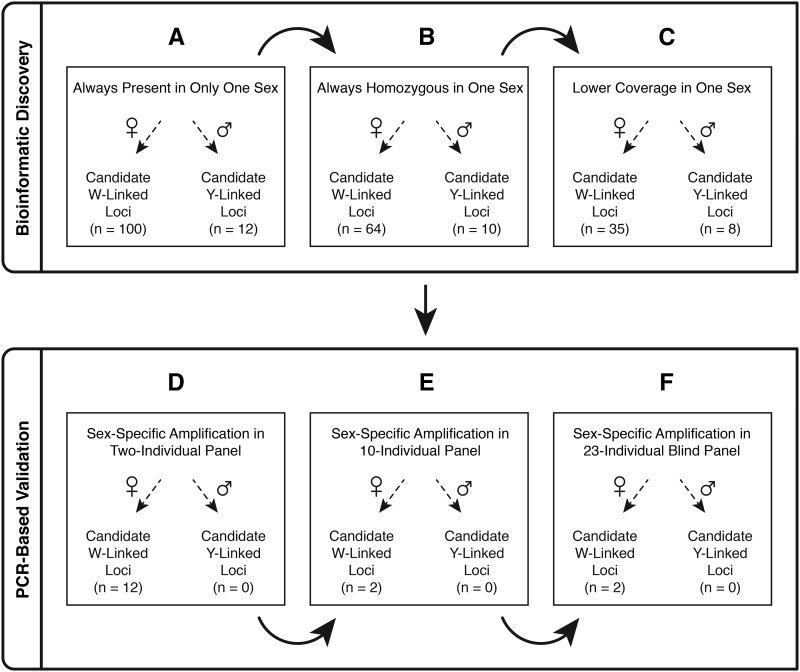
Pipeline for identifying putative sex-linked loci and for testing the competing hypotheses of female-heterogametic (ZW) *vs.* male-heterogametic (XY) sex determination in Cryptobranchidae. If a locus is found only on the heterogametic sex chromosome, then (A) it will only be found in one sex, (B) there will only be one allele because the individual is haploid (hemizygous) at that locus, (C) that locus will have approximately half the read depth of other loci because it is haploid, (D) sex-linked loci should identify known-sex individuals in two-individual panels, (E) 10-individual panels, and (F) 23-individual panels.

## Materials and Methods

We implemented a two-part strategy to discover and validate candidate sex-linked regions of the *Cryptobranchus* genome (Figure 2). Because of uncertainty about whether cryptobranchid salamanders have ZW or XY sex determination, we conducted analyses agnostically for both scenarios and tested three specific hypotheses about expected patterns of genetic variation in males and females under models of XY or ZW sex determination. We first used a series of bioinformatic analyses to quantify patterns of sex-specific presence and absence of loci, to parse heterozygous loci from apparently homozygous loci (some of which may be hemizygous), and to estimate relative depths of read coverage across candidate sex-linked loci (Figure 2A–C). Next, we used PCR-based assays in additional individuals to validate the sets of candidate male- and female-specific loci that were identified bioinformatically. Candidate sex-linked loci (35 female-specific candidates and eight male-specific candidates) identified in 20 reference individuals were screened by PCR in increasingly large and diverse sets of individuals (Figure 2D–F).

We obtained tail tissue or blood samples from known-sex *Cryptobranchus alleganiensis* individuals (*n* = 66) sampled from wild populations and from captive collections at the Saint Louis Zoo. We also obtained blood samples from known-sex *Andrias davidianus* (*n* = 6) and *A. japonicus* (*n* = 9) individuals from the Saint Louis Zoo, the Detroit Zoo, the California Academy of Sciences Steinhart Aquarium, and the Smithsonian National Aquarium. We used 20 known-sex *Cryptobranchus* individuals from two separate tributaries of the White River in Missouri and Arkansas (nine females and 11 males, as determined by necropsy and/or observation of gametes) for our initial reduced representation genome sequencing by ddRADseq. All other individuals were sexed by visual examination during the breeding season or by either laparoscopy or ultrasound. High molecular weight genomic DNA (gDNA) was extracted from all individuals using DNeasy silica column kits (QIAGEN). We quantified the resulting gDNA with a Qubit2.0 Fluorometer (Invitrogen) and confirmed its integrity by 2% agarose gel electrophoresis at 110 V for two hr.

### Bioinformatic discovery of candidate sex-linked loci

The extremely large size of the *Cryptobranchus* genome, and the lack of existing genomic resources for this genus, necessitated the use of reduced representation sequencing methods to identify sex chromosomes. We used ddRADseq because it offered the flexibility to select different RE combinations and size-selection windows that can produce varying amounts of genomic coverage. To optimize ddRAD library preparation in this massive genome, we performed test RE digestions for two *Cryptobranchus* individuals from the White River in Missouri, followed by empirical fragment analysis. We estimated numbers of fragments per individual under three different size selection windows and 12 different RE combinations. For each RE combination, we performed single-enzyme digests and the combined double-enzyme digests, and we quantified these test fragment length distributions with a Bioanalyzer 2100 high-sensitivity DNA system (Agilent Technologies). We then calculated the estimated numbers of sequenceable ddRAD loci across size selection windows of 300 ± 30 base pairs (bp), 400 ± 40 bp, and 500 ± 50 bp. Based on these tests, we selected *Eco*RI (3′) and *Sph*I (5′) with a fragment size selection window of 450–550 bp for downstream library preparation. These parameters were estimated to yield ∼350,000 unique loci per individual (significantly fewer than any other RE combination). We increased the amount of input gDNA from 50 ng (Peterson *et al.* 2012) to 3 μg per individual to retain sufficient quantities of post-bead-cleaned product for adapter ligation.

We generated ddRAD libraries with four sets of five individuals using 5 bp inline barcodes and 6 bp Illumina indices. We size-selected each pool of five individuals in its own lane of a Pippin Prep cartridge (Sage Science) with the “tight” protocol in the range of 518–634 bp (accounting for lengths of Illumina adapters). Size-selected products were pooled into two sets of 10 individuals, bead-cleaned, and amplified by PCR for 8 cycles with Phusion high-fidelity DNA polymerase (New England Biolabs). Bead-cleanup was performed with Dynabeads (ThermoFisher) and Agencourt AMPure XP beads (Beckman Coulter, Inc.). Final libraries were sequenced on two Illumina HiSeq2500 lanes in Rapid Run mode with paired-end 150 bp reads (utilizing C-bot clustering and a 10% *Phi*X DNA spike-in). Illumina sequencing was performed at the Florida State University School of Medicine Core Facility.

This library preparation protocol resulted in strand-specific sequencing because the PCR primers for fragment amplification select for fragments with *Sph*I cut sites at the 5′ end and *Eco*RI cut sites at the 3′ end. Each sequenced fragment was represented by 150 bp sequences at the 5′ and 3′ ends and a central unsequenced region of unknown length (between 150–250 bp). We used custom bash scripts (Files S1) to concatenate reads from the 5′ ends of fragments (R1 of an Illumina read pair) with the reverse complement of reads from the 3′ ends of fragments (R2 of an Illumina read pair), recapitulating the original orientation in the genome. We checked for optical duplicate reads using the MarkDuplicates function in the Picard Toolkit (https://broadinstitute.github.io/picard/). We demultiplexed the raw, stitched reads by individual in stacks v1.46 (Catchen *et al.* 2013) with the process_radtags function, allowing one mismatch between observed and expected barcodes. We retained only reads with appropriate RE cut sites at both ends and required reads to have a mean Phred quality score greater than 20 over 45 bp sliding-window intervals (amounting to the following settings for the stacks process_radtags algorithm: –renz_1 sphI –renz_2 ecoRI -c -q -r -D -w 0.15 -s 20 –barcode_dist_1 2).

We tested several assembly parameters for the range of nucleotide variation between alleles at a given locus (ustacks -M = 4, 10), the minimum depth of sequencing coverage across loci (ustacks -m = 3, 10), and variation between alleles across the set of individuals (cstacks -n = 0, 16). We used sstacks to match individual loci back to the full locus catalog, and we reconstructed haplotypes across all loci for all 20 individuals with genotypes (-r 1 -m 3). We identified parameter settings for ustacks (-m 3 -M 4 -N 10) and for cstacks (-n 0) that optimized the recovery of putatively orthologous, single-copy regions in the *Cryptobranchus* genome. These assembly parameters allowed us to confidently call variable sites across loci and to determine whether individuals were homozygous or heterozygous at a particular locus. Any locus with more than two haplotypes in any of the 20 reference individuals was excluded from further analyses. We used custom bash scripts (File S1) to parse the locus catalog into sets of putatively male- and female-specific loci based on presence/absence, zygosity, and relative depth of coverage (Figure 2A–C, File S2).

### PCR-based validation of candidate sex-linked loci

Because ZW and XY sex determination systems are mutually exclusive, we predicted that either all of the male-specific candidate loci or all of the female-specific candidates would co-amplify by PCR in both sexes, rejecting one of these alternative hypotheses about the mode of sex determination in cryptobranchid salamanders. All candidate loci were subjected to successive rounds of PCR validation in increasing numbers of known-sex individuals (Figure 2D–F). First, an initial PCR validation step was performed in one male and one female hellbender from the White River for which sex was definitively known from post-mortem examination of gonads. Loci with sex-specific amplification in the two-individual panel were then tested in a 10-individual panel of six *Cryptobranchus* individuals from the White River drainage in Missouri, two individual *Cryptobranchus* from the Blue River in Indiana, and two *Andrias davidianus* (a total of five males and five females). Loci with sex-specific amplification in the 10-individual panel were then tested in a blind trial with 23 known-sex *Cryptobranchus* from several rivers in Missouri (Gasconade, Big Piney, Niangua, Meramec, and Current Rivers). Sexes of individuals in the blind trial were determined by external morphology during the breeding season by J.T.B., but were unknown to P.M.H. before the trial. To further validate candidate sex-specific loci in more divergent populations of *Cryptobranchus* and in both species of *Andrias*, we tested any retained candidate loci across 18 known-sex *Cryptobranchus* from the Licking River watershed in Kentucky and in a set of four additional *Andrias davidianus* and nine *A. japonicus* (Table S1).

Oligonucleotide primers (Table S2) were designed for eight putative Y-linked loci and 35 putative W-linked loci in BatchPrimer3 (You *et al.* 2008) using default settings except for: primer length (minimum 23 bp, optimum 30 bp, maximum 33 bp), maximum difference in melting temperature between forward and reverse primers (5°), and optimal amplicon fragment length (minimum 375 bp, optimum 500 bp, maximum 550 bp). We used an optimal primer length of 30 bp to ensure primers would bind nonrandomly in the complex *Cryptobranchus* genome. An autosomal positive control locus was designed for a 756 bp region of the 18S ribosomal RNA (rRNA) gene in *Cryptobranchus*. Although nuclear-encoded rRNA may exist in multiple copies throughout the genome, this marker is a suitable positive PCR control because amplification of at least one 18S copy indicates a successful PCR reaction. PCRs were carried out in 20 μL volumes [200 μM dNTPs, 0.5 μM forward and reverse primers, 109 ng gDNA, 0.4 units Phusion DNA polymerase (New England BioLabs)] with a “hotstart” initial denaturation at 98° for 30 sec, followed by 40 cycles of 98° denaturation for 10 sec, a locus-specific annealing temperature for 20 sec (detailed in Table S2), and a 72° extension for 30 sec, followed by a final 72° extension for 10 min. PCR products were stained with 2X EZ-Vision Two dye (VWR Life Science) and visualized on 1.3% agarose gels run for 45–75 min at 110 V.

### Data Availability

Table S1 details the individuals examined. Table S2 details the candidate sex-linked ddRAD loci and PCR primers. Table S3 summarizes the gel images. File S1 contains example bash scripts. File S2 details candidate locus identification by presence/absence, zygosity, and depth of coverage. Figure S1 outlines the ddRAD protocol. Figure S2 summarizes locus assembly results across individuals. Figure S3 shows the effect of individual sampling on numbers of candidate sex-linked loci. Figures S4–S8 contain gel images for validation tests. Supplemental files and Illumina data are available in this study’s Figshare accession: https://gsajournals.figshare.com/s/bd1a11f0e64a4622ff43. Demultiplexed and stitched Illumina data are also accessioned in the NCBI Short Read Archive (PRJNA553239: SRR9655295–SRR9655314). All work was conducted in accordance with applicable institutional guidelines for animal welfare under Saint Louis Zoo IACUC protocols 2009-04 and 2010-07 (to P.M.H.). Supplemental material available at FigShare: https://doi.org/10.25387/g3.8060006.

## Results

### Bioinformatic discovery of candidate sex-linked loci

Across all 20 *Cryptobranchus* individuals used for ddRADseq, we obtained 163,104,028 read pairs. After initial demultiplexing, quality filtering, and RE cut site verification and truncation, we retained 113,835,666 read pairs totaling 33,809,192,802 bp (after trimming in-line adapter sequences). On average, males and females had roughly equal numbers of retained reads per individual, but there was significant variation in the numbers of reads per individual (Table 1). The nine females had on average 5,925,697 read pairs per individual (range 1,671,803–10,897,888 reads) and the 11 males had on average 5,500,399 read pairs per individual (range 2,980,522–8,934,375 reads). The Picard Toolkit (https://broadinstitute.github.io/picard/) identified zero reads as duplicates. We used the stacks pipeline (Catchen *et al.* 2013) to assemble loci and make preliminary haplotype calls for each individual (ustacks), to assemble a catalog relating all loci across all individuals (cstacks), to find catalog matches and call single-nucleotide polymorphisms for each individual (sstacks), and to call haplotypes across all individuals (genotypes). There was significant variation in the number of ustacks loci assembled for each individual (147,468–387,597), with a general positive (but asymptotic) correlation between the number of input reads and the number of ustacks loci (Figure S2). Across all ustacks loci for all 20 individuals, we assembled a catalog of 1,590,599 unique cstacks loci. Not all loci were present for all individuals, and in fact, all individuals had loci that were shared with nearly every possible combination of other individuals. This complex situation reflected, in part, a lack of saturation of loci due to uneven sequencing coverage across individuals. However, some of this variation in the overlap of loci across individuals reflected cryptic patterns of sex-linkage.

**Table 1:** Details of ddRAD sequencing in 20 known-sex *Cryptobranchus alleganiensis bishopi*

Individual	Barcode	Sex	Filtered Reads	Ustacks Loci	Mean Depth
C38AF	ACTGG	Female	10,897,888	387,597	27.51
C39AF	ACTTC	Female	8,453,283	364,016	22.69
C91CF	ATACG	Female	9,888,747	362,344	26.73
C92DF	ATGAG	Female	1,671,803	147,468	10.94
C93DF	ATTAC	Female	2,609,406	194,087	13.03
C100EF	CATAT	Female	5,058,754	283,782	17.39
C104EF	CGAAT	Female	3,495,437	229,634	14.82
C109EF	CGGTA	Female	4,170,971	257,133	15.81
C110EF	CGTAC	Female	7,084,986	324,687	21.34
C31AM	GCATG	Male	8,668,485	366,678	22.46
C32AM	AACCA	Male	3,243,953	260,992	11.47
C33AM	CGATC	Male	2,980,522	251,412	10.93
C34AM	TCGAT	Male	5,346,185	311,318	16.22
C35AM	TGCAT	Male	4,507,062	290,538	14.65
C36AM	CAACC	Male	7,184,657	350,200	19.35
C89CM	GGTTG	Male	5,613,817	321,310	16.45
C94DM	AAGGA	Male	8,934,375	351,762	24.31
C97DM	AGCTA	Male	4,946,750	315,847	14.62
C101EM	ACACA	Male	5,932,130	322,649	17.38
C108EM	CGGCT	Male	3,146,455	216,458	14.13

When comparing only a few individuals of each sex, many loci were present uniquely in one sex and absent in the other sex, and this pattern held for both males and females. As greater numbers of individuals from each sex were compared, the numbers of putatively sex-specific loci dropped precipitously (Figure S3). After comparing loci for all nine female and 11 male *Cryptobranchus*, we retained a set of 12 loci present in all males and absent in all females (putatively Y-linked) and a set of 100 loci present in all females and absent in all males (putatively W-linked) (Figure 2A). To reduce the number of loci involved in PCR screening steps, we excluded any sex-specific candidate loci for which any individual of the putatively heterogametic sex had more than one haplotype identified, resulting in 10 male-specific candidate loci and 64 female-specific candidate loci (Figure 2B). Because W- or Y-linked loci should have roughly half the depth of coverage of autosomal loci, we applied read-depth filtering to further reduce the sets of candidate sex-linked loci to eight male-specific candidate loci and 35 female-specific candidate loci (Figure 2C).

### PCR-based validation of candidate sex-linked loci

**Figure 3:**
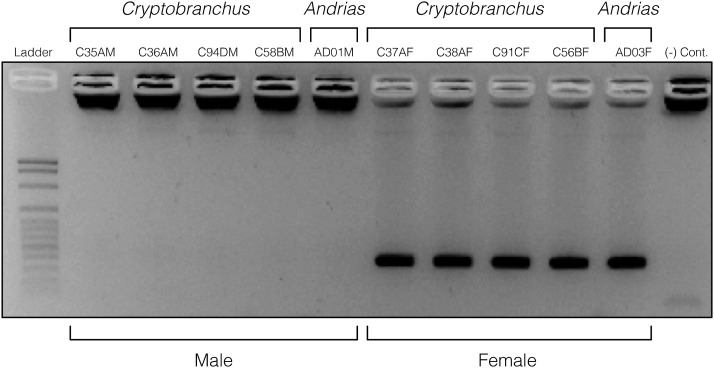
Sex-specific amplification of a putative W-linked marker (candidate locus 1024220) in the 10-individual panel of *Cryptobranchus* and *Andrias*. A 100 base pair ladder and a negative PCR control are shown in the leftmost and rightmost lanes, respectively. This image was exposed manually and pixels were inverted on the gel imaging system at the time of capture, but contrast and exposure have not been subsequently modified. Dark blobs in the male and control wells are loading dye bound to non-migrating DNA. The gel image has been cropped at the edges but all lanes are shown.

After an initial round of two-individual PCR tests in *Cryptobranchus*, we retained zero putatively male-specific loci (all of these loci co-amplified in both sexes) and 12 putatively female-specific loci which amplified only in females (Figure 2D, Figure S4). A subsequent screening of the 12 putatively female-specific loci using a 10-individual panel of *Cryptobranchus* and *Andrias* revealed two putatively W-linked loci (1024220 and 1102805) that consistently amplified in all females and never amplified in any males (Figure 2E, Figure 3, Figure S5). A blind trial with 23 known-sex *Cryptobranchus* from additional populations in Missouri demonstrated successful amplification only in the known-females for both W-linked markers, while the 18S rDNA positive control successfully amplified in all individuals (Figure 2F, Figure S6). Further testing of these two putative W-linked markers in *Cryptobranchus* individuals from Kentucky confirmed that these loci successfully amplify only in females for this divergent lineage of hellbenders (Figure S7). Each of the two putative W-linked markers consistently amplified in females of both species of *Andrias* (Figure S8), with the exception of one individual that had degraded gDNA. Table S3 summarizes all gel results.

## Discussion

Our results strongly support the hypothesis of female heterogamety in cryptobranchid salamanders. Identification of consistent female-specific amplification of two loci and the lack of male-specific amplification lead us to reject male-heterogamety in the family Cryptobranchidae. Our results corroborate recent ddRADseq-based findings of a ZW sex determination system in *Andrias* by Hu *et al.* (2019), and expand on their results by providing strong evidence for a ZW system in the common ancestor of *Cryptobranchus* and *Andrias* about 60 million years ago when these two genera likely diverged (Zhang and Wake 2009; Liang *et al.* 2019). This study also demonstrates the power of reduced representation genome scans for identifying sex-linked genes in organisms that lack pre-existing genetic resources. Our results further suggest that genome size may not necessarily be a limiting factor in generating informative genome-scale data to answer evolutionary questions in salamanders [see also Nunziata *et al.* (2017); Murphy *et al.* (2018); Weisrock *et al.* (2018); Hu *et al.* (2019)].

### Investigating sex determination in a massive genome

Comparing multiple, known-sex individuals is an important aspect of sex-specific locus detection, and contrasts drawn from greater numbers of individuals reduce the numbers of putatively sex-linked loci which must be screened by PCR in downstream steps. In our case with cryptobranchid salamanders, the number of candidate loci identified by analyzing a single representative of each sex decreased by roughly an order of magnitude each time that we doubled the number of individuals of each sex under consideration (comparing two, four, or eight individuals of each sex) (Figure S3), similar to findings from Gamble and Zarkower (2014) (their Figure 5). In more challenging cases where sequencing coverage is particularly uneven across individuals, relaxing the requirement that loci are present for all individuals of a given sex (but still requiring absence in all individuals of the opposite sex) could potentially lead to greater detection of putatively sex-linked loci (Stovall *et al.* 2018). As a cautionary note, we stress that for RAD-based studies searching for sex-linked markers, the sexes of individuals must be known with absolute certainty, especially for species with large genomes which will have large numbers of loci under consideration.

We expected the massive cryptobranchid genome to pose significant challenges for generating genomic data in these species and our ability to screen and validate putative sex-specific loci. Across multiple combinations of REs and size selection windows, we found that nearly all combinations would produce far too many loci (as many as four million loci per individual) to achieve adequate multiplexing of individuals on the Illumina platform. Using *Sph*I and *Eco*RI and size selection between 450 and 550 bp, we expected to produce ∼350,000 loci per individual, very much in line with the empirical numbers of ustacks loci which we assembled in the highest-coverage individuals. Although a significant proportion of these loci had affinities to known transposable elements, filtering out loci with greater than two haplotypes or depth of coverage greater than three standard deviations above the mean for an individual effectively removed most tranposable element-related loci from consideration. Future efforts to sequence larger genomic regions flanking these sex-linked loci will better characterize these chromosomal regions and facilitate more in-depth studies of the gene regions residing on cryptobranchid sex chromosomes.

### Implications for applied conservation in Cryptobranchidae

These W-linked markers provide a robust genetic sex assay for cryptobranchid salamanders, akin to those already widely employed in avian taxa (Ellergren 1996), mammals (Taberlet *et al.* 1993), and squamate reptiles (Rovatsos and Kratochvíl 2017), and could be a massive boon for conservation and repatriation efforts in these imperiled salamanders. Researchers and conservation biologists may now accurately determine sex for entire clutches to inform captive breeding efforts or to test expectations of male-biased adult sex ratios in species with female heterogamety (Pipoly *et al.* 2015). This molecular sex diagnostic could also enable studies into whether adult cryptobranchids undergo environmentally induced sex reversal by searching for a mismatch between morphological and genotypic sex (Hayes *et al.* 2010; Xu *et al.* 2015; Tamschick *et al.* 2016; Hu *et al.* 2019).

Although the W-linked markers identified here are robust across all *Cryptobranchus* populations tested (spanning most of the major lineages across the geographic distribution) and in both species of *Andrias*, it is possible that mutations in primer binding sites, or PCR failure in general, could result in a lack of amplification of the W-linked markers. In these cases, one would be led to an incorrect inference that individuals were male. The converse situation (W-linked loci spuriously amplifying in males) is extremely unlikely. Using multiple W-linked markers for sex diagnosis and employing a positive PCR control (*e.g.*, 18S rRNA) should lead to increased confidence in PCR-based sex diagnosis.

### Conclusions

Using ddRAD genome scans in known-sex hellbenders, we demonstrate that both *Cryptobranchus* and *Andrias* possess a ZW system of female heterogamety and that homologous loci on the W sex chromosomes of these two genera have likely been maintained as sex-linked for nearly 60 million years. These findings will be important for tracing the evolution of sex chromosome turnovers within salamanders. This work also has significant implications for applied conservation efforts with cryptobranchid salamanders. Whereas it has previously been difficult to reliably distinguish male from female cryptobranchids on the basis of morphology, our study has developed a universally effective PCR-based assay for sex in this imperiled salamander family. These methods for interrogating genetic sex determination systems are also broadly applicable in other organisms with large genomes and homomorphic sex chromosomes. The W-linked loci described here may enable new and important research and conservation directions for cryptobranchid salamanders, and set the stage for broader-scale comparative evolutionary genomic research across amphibians.
